# The impacts of road traffic on urban air quality in Jinan based GWR and remote sensing

**DOI:** 10.1038/s41598-021-94159-8

**Published:** 2021-07-30

**Authors:** Qi Wang, Haixia Feng, Haiying Feng, Yue Yu, Jian Li, Erwei Ning

**Affiliations:** 1 School of Transportation and Logistics Engineering, Shandong Jiaotong University, Jinan, 250023 China; 2 Beijing Technology and Business University, Beijing, 100101 China

**Keywords:** Environmental impact, Environmental impact

## Abstract

Traffic congestion and smog are hot topics in recent years. This study analyzes the impacts of road traffic characteristic parameters on urban air quality quantitatively based on aerosol optical thickness (AOD) and geographical weighted regression (GWR) models, including the road network density, road area occupancy, intersection number, and bus network density as main factors. There are some major research findings. Firstly, there exists a strong positive correlation between the peak congestion delay index (PCDI) and air quality, the correlation has R^2^ values of up to 0.4962 (R 0.70). Secondly, GWR refines the local spatial changes in the AOD and the road parameters, and the correlation R^2^ based GWR model all above 0.6. The correlation between AOD and the road area occupancy was the highest, and the correlations with the bus network density and the intersections number were higher than that with the road network density. Thus, bus route planning, bus emission reduction, road network planning, and signal timing (at intersections) have a greater impact on air quality than other policy, especially in areas with traffic jams. The results of this study could provide theoretical support for traffic planning and traffic control, and is promising in practice.

## Introduction

As the largest developing country, China's air quality has always been a focus of research. Air pollution is formed by a complex set of mechanisms, and various factors have been demonstrated to have an impact on it, including meteorological conditions, socio‐demographic characteristics, built environment factors, etc., among them the vehicle exhaust pollution paid more and more attention to. According to the pollutant analysis results released by Beijing, Jinan, Hangzhou, vehicle exhaust has surpassed coal as the main source of urban air pollution (especially PM2.5). By June 2020, the number of motor vehicles in China had reached 360 million, and traffic congestion had become the norm in many Chinese cities. As a result, the contribution rate of vehicle exhaust pollution to air quality will continuously increase^1,2^ (Liu et al. 2018; Huang et al. 2020a). Its contribution to PM2.5, volatile organic compounds (VOC), etc., were experimentally analyzed^3–8^ (Kazuo et al. 2019; Lin et al. 2020; Oish et al. 2019; Pathak et al. 2020; Wang et al. 2018;Watson et al. 2001). The influences of the traffic characteristics, traffic sources, traffic flow states, road grade, vehicle type, fuel, terrain, meteorological conditions, and spatial–temporal heterogeneity on exhaust emissions were studied^9–17^(Abdull et al. 2020; Bae et al. 2018; Beddows et al. 2020; Jeong et al. 2019; Huang et al. 2020b; Li et al. 2018; Lin et al. 2019; Liu et al. 2019; Pratama et al. 2019; Zhang et al. 2021). Traffic simulations, the OMG volume-source model, cellular automata, sensitivity analysis, and the fault tree model have also been used to study exhaust emissions, diffusion, and their influence on air pollution^18–23^ (Chen et al. 2020; Ibarra-Espinosa et al. 2020; Matzoros et al. 1992; Mdziel et al. 2020; Xu et al. 2020; Wang et al. 2019). Few studies were focus on the impacts of road network traffic characteristics (e.g., road density, intersection, and bus network density) on air quality, especially the road network traffic characteristics have the spatial heterogeneity, but there is spatial heterogeneity in air pollution and the processes of producing air pollution. It had been proved that GWR model considering the local effects of spatial objects (i.e., the spatial heterogeneity) was an effective tool to describe spatial heterogeneity^24–26^(Zhao et al. 2017; Fotheringham et al. 2017, 2019).

The retrieval of the air pollution status based on remote sensing data not only makes up for the lack of observation data, but also reflects the spatial distribution characteristics of the air pollution, so remote sensing inversion has become an important method for studying air pollution. Previous studies have shown that there is a strong correlation between the aerosol optical thickness (AOD) and the concentration of near surface particles, and the AOD product of MODIS (Moderate Resolution Imaging Spectroradiometer) is the most widely used in air pollution research^27–29^ (Sathe et al. 2019; Tao et al. 2012; Wei et al. 2021). The objective of this study is to quantitatively analyze the impacts of road traffic characteristics on urban air quality based on their spatial heterogeneity.

## Materials and methods

### Study area

**Figure 1:**
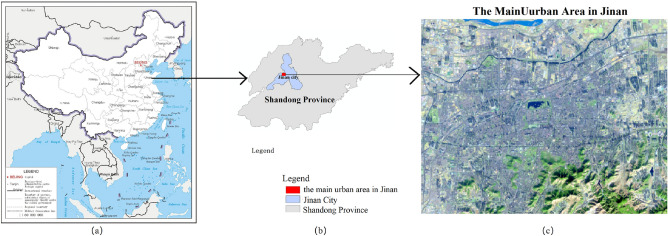
Location of the study area. A is the map of China(http://bzdt.ch.mnr.gov.cn/browse.html?picId=%224o28b0625501ad13015501ad2bfc0291%22) , and B and C show the location of study area (the main urban area in Jinan) and the Landsat-8 OLI image in study area. Map created in ArcMap 10.5 of the Environmental System Resource Institute, Inc. (https://www.esri.com/software/arcgis/arcgis-for-desktop). Boundaries made with free vector data provided by National Catalogue Service for Geographic Information (https://www.webmap.cn/commres.do?method=dataDownload).

The main urban area in Jinan (non-administrative boundaries, see Fig. 1 was taken as the study area. There are two reasons: severe haze and traffic congestion. Jinan has been ranked among the cities in China with the worst smog problems, and its annual average PM2.5 concentration was greater than 90 μg/m^3^ in 2016 and 2017. The other reason why Jinan was selected as the study area lay in its serious traffic congestion. The Traffic Analysis Report published by AMap showed that Jinan ranked No. 1 among the cities in China in terms of commuter peak congestion in 2016 and 2017, and the peak road network congestion delay index (PCDI) reached 2.28 and 2.14 in 2016 and 2017, respectively. Traffic congestion was eased in 2018 and 2019. In the first quarter of 2020, Jinan became the "first traffic jam" in China again. As the capital of Shandong Province, Jinan is located in the western-central part of Shandong Province, south of Mount Tai, and crossing the Yellow River in the north.

In this study, data collected on December 26, 2017, was chosen for research for the following reasons:(1) Traffic conditions: on Dec 26, 2017, the traffic congestion in Jinan was severe and all of the major traffic arteries, such as Jingshi Road, Beiyuan Road, and the main roads in Jinan, were experiencing longer severe congestion; (2) Weather: it was sunny with northeast winds of < 3 m/s, which is favorable for remote sensing data; (3) Air quality: the air in Jinan was mildly polluted, with a mean PM2.5 value of 85 μg/m^3^. The traffic, weather, and air quality in the study area on December 26, 2017, were consistent with the study requirements.

### Data description

**Figure 2:**
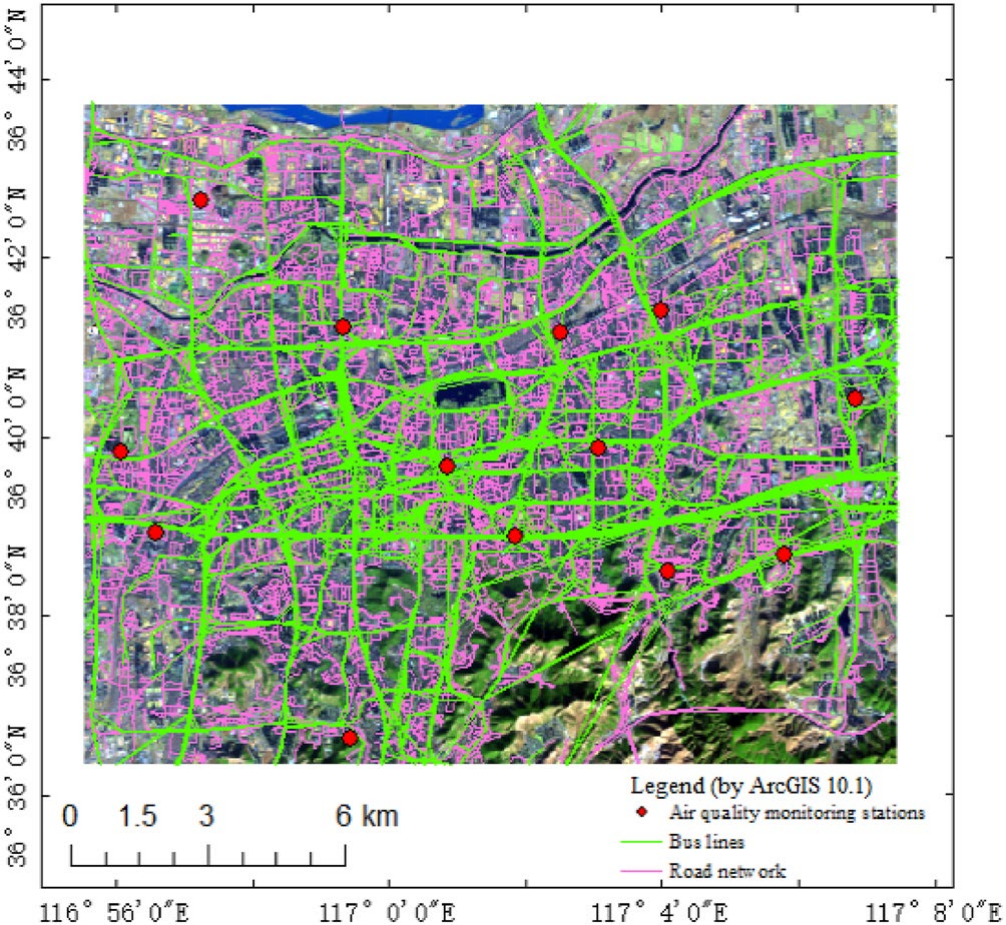
Air quality monitoring stations, bus lines, and road network in the study area. The red dots are the air quality monitoring stations; the green lines are the bus lines and the pink lines are road network. Map created in ArcMap 10.5 of the Environmental System Resource Institute, Inc. (https://www.esri.com/software/arcgis/arcgis-for-desktop).

In total, monitoring data from 11 air quality monitoring stations in the study area (https://www.aqistudy.cn) and two stations that were deployed by two research teams were utilized. The distribution of the monitoring stations is shown in Fig. 2.

Two types of remote sensing data were used in this study: MCD19A2 AOD products and landsat8 OLI images. MCD19A2 is the official 1 km resolution AOD products of MODIS, and it is produced using the aerosol algorithm in MAIAC (the multi-angle atmospheric correction algorithm). Compared with the 10 km and 3 km resolution MOD04 aerosol products, the MCD19A2 has a higher resolution (study area located in h27v05). The column number of the Landsat8 OLI image of Jinan was 12,235. Both datasets were collected on December 26, 2017.

Roads less than 3 m wide were excluded from the road network in the study area. The network of public transport routes in the study area is shown in Fig. 2.

As an evaluation index of urban congestion degree, the Peak Congestion Delay Index (PCDI, typically at 7:00–9:00 in the morning and at 17:00–19:00 in the evening.) is the ratio of the average actual travel time of urban residents to the travel time in free state, which is a representation of a city's traffic operations. The greater of PCDI, the more congested the traffic, the slower the driving speed, and the more exhaust emission. The PCDI was obtained from China's Major Urban Transport Report released by AMap.

## Methods

### Air quality distribution based on MCD19A2

**Figure 3:**
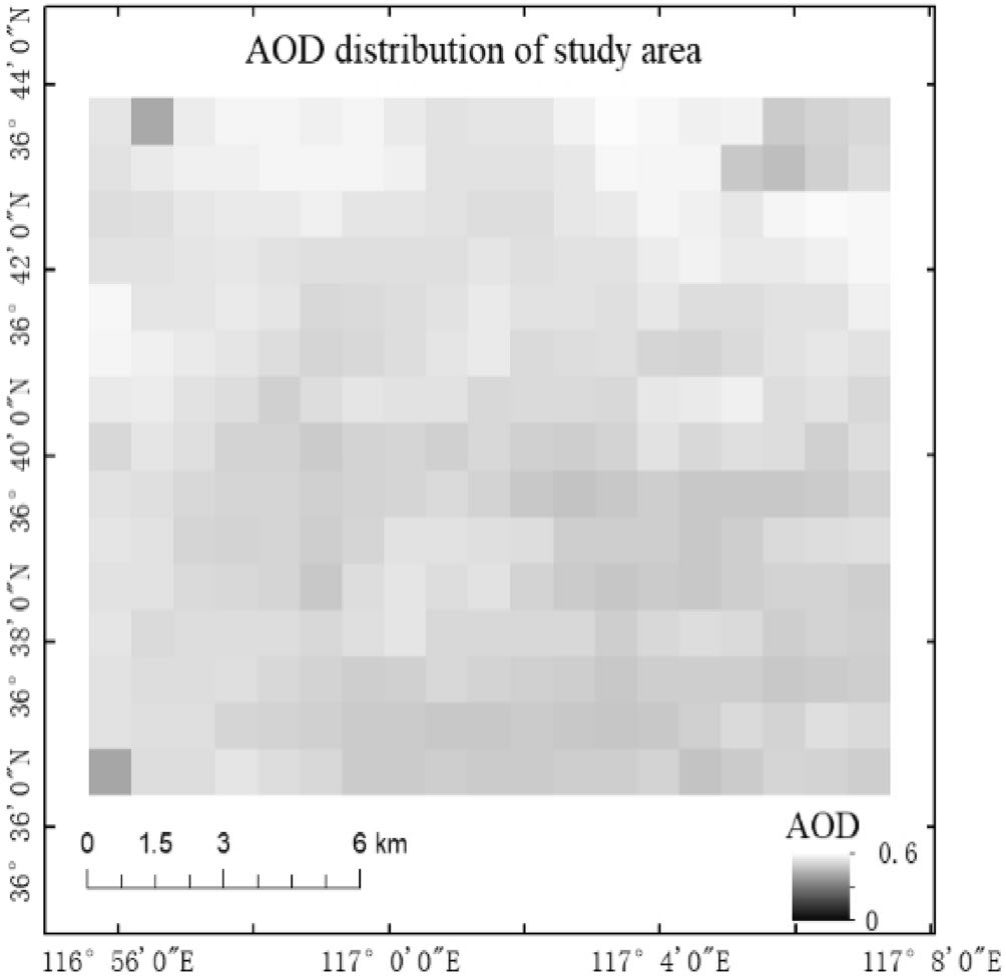
AOD distribution in the study area. AOD reflects the air quality. Generally, the brighter in image is, the higher the AOD value is, and the more serious the air pollution is. Map created in ArcMap 10.5 of the Environmental System Resource Institute, Inc. (https://www.esri.com/software/arcgis/arcgis-for-desktop).

The MCD19A2AOD products for the different orbits (there are 4 tracks in the daily AOD data) were combined into the daily AOD, and the AOD in the study area is shown in Fig. 3.

### Gridding

Using the gridding tool, the study area was divided into 1 km × 1 km grids, with less than 1 km remaining on the northern and eastern sides of the study area being excluded from the gridding. The AOD, road network density, road area ratio, and number of intersections in each grid were calculated.

### GWR-based analysis of the impact of the road network traffic characteristics on the urban air quality

Geographically weighted regression (GWR) is a spatial analysis technique that is widely used in geography and related disciplines involving the analysis of spatial patterns, and it can be used to quantify spatial heterogeneity. It has higher accuracy than regression model because the local effects of spatial objects (i.e., the spatial heterogeneity) are taken into account.

$${\text{y}}_{i} = \beta_{0} (u_{i} ,v_{i} ) + \sum\limits_{k = 1}^{p} {\beta_{k} (u_{i} ,v_{i} )x_{ik} } + \varepsilon_{i},$$

where $\beta_{{0}} ({\text{u}}_{i} ,v_{i} )$ is the coordinates of sample point ${\text{i}}$; $\beta_{{\text{k}}} (u_{i} ,v_{i} )x_{ik}$ is the kth regression parameter at sample point I; and $\varepsilon_{{\text{i}}}$ is the error correction term.

The GWR analysis was based on the spatial correlation test of AOD distribution in the study area. The spatial autocorrelation analysis tool in ArcGIS (the Moran's Index I) was used for the analysis. Moran's index I is mainly used to measure the spatial distribution characteristics of the data throughout the entire region.

The Moran's index I distributions of AOD was 0.49 in the study area, the distributions of the AOD was closely correlated, had obvious spatial clustering characteristics, and was heterogeneous, which makes them suitable for GWR analysis.

### Correlation analysis

**Figure 4:**
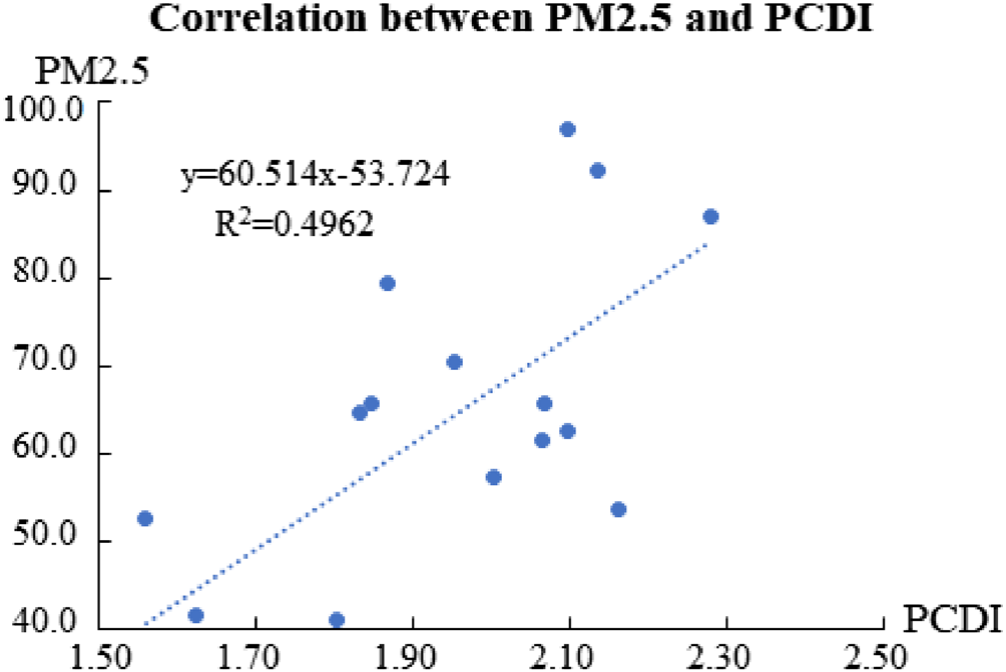
Correlation between PM2.5 and PCDI. This figure shows the correlations between PCDI and PM2.5, and which shows PCDI has high positive correlation with PM2.5.

The correlation between the PCDI in each quarter for the past five years (2016 to Q1 2020) and the PM2.5 (the average values of each quarter in Jinan) was analyzed, and the fitting diagram is shown in Fig. 4.

The correlations between AOD and the gridded density road network, the road area occupancy, the number of intersections, and the network density of the bus lines were all analyzed (linear regression).

## Results

### Traffic and air quality

As seen in Fig. 4 the correlation coefficients between the PCDI and the PM2.5 has R^2^ values of up to 0.4962 (R 0.70). The data were paired separately and the F Test was conducted in the two groups of data when p < 0.05, i.e., the samples were variance congruent. Then the t-Test was conducted using the two-sample equal variance hypothesis. With a = 0.05, all p < 0.05, that was, the fitted linear equation passed the significance test. The results demonstrate PM2.5 is positively correlated with PCDI. According to the source analysis of PM2.5 in Beijing, Jinan, and Hangzhou, exhaust gases surpassed coal combustion as the main source of PM2.5 pollution in cities, that is, traffic (exhaust) has a large impact on urban air quality, especially in traffic jams. However, the correlations between AOD and the gridded density road network, the road area occupancy, the number of intersections, and the network density of the bus lines (linear regression) were all low, so the GWR model considering spatial heterogeneity was used.

### Single-parameter impact analysis

The AOD model with road network density, road area occupancy, number of intersections, and bus network density were constructed separately based on GWR. The contrast between the input parameters in the GWR output tool is shown in Table 1.

**Table 1:** GWR model input comparison table.

Dependent field	Explanatory field	R^2^	R^2^ adjusted
AOD	Road network density	0.6075	0.4814
	Road occupancy rate	0.6484	0.5155
	Number of intersections	0.6162	0.4912
	Density of the public transport network	0.6304	0.5075

As can be seen from Table 1, the AOD based on the GWR model analysis was closely correlated with the road network density, road area occupancy, and the number of intersections. That is, AOD has strong heterogeneities, so the GWR model was used to refine the characterization of the AOD distribution and the local spatial variation in the road network in the study area. The regression parameters for each variable were positive or negative, i.e., each factor had a facilitating or inhibiting impact on air quality in the different regions. However, based on the median of the regression coefficients for each variable, it is clear that the road network density, road area occupancy, and number of intersections had significant impacts on the regional AOD, and they were positively correlated.

The road area occupancy was exhibited the highest correlations with AOD, i.e., during traffic congestion, the road occupancy area had the greatest impact on the air quality in the region. AOD was better correlated with the density of the bus route network than with the density of the road network, which means that buses had a greater impact on the urban AOD, and reducing pollution from buses is important for reducing urban AOD concentrations. AOD was more significantly correlated with the number of intersections than with the density of the road network, i.e., the setting of the traffic lights (traffic signals) within the regional road network. The number of intersections and the network of bus routes had significant impacts on the exhaust emission (i.e., the regional air quality).

### Parameter autocorrelation analysis

In this study, the road network density, the road area occupancy, the number of intersections, and the bus route network density had significant impacts on the air quality in the corresponding areas. To avoid multicollinearity among the variables, the correlations between the four parameters themselves were analyzed before constructing the multi-parameter GWR model Table 2.

**Table 2:** Correlation coefficient R^2^ of parameters.

	Road network density	Road occupancy rate	Number of intersections	Density of the public transport network
Road network density	1			
Road occupancy rate	0.924	1		
Number of intersections	0.8999	0.7891	1	
Density of the public transport network	0.2392	0.2463	0.2685	1

As can be seen from Table 2, the three parameters, including the road network density, the road area occupancy, and the number of intersections, were significantly correlated. In particular, the correlation coefficient between network density and road area occupancy was 0.924, but the correlation between road area occupancy and intersections was weaker (0.7891). The correlations between the bus route network density and the other three parameters were less significant (less than 0.3). Thus, road area occupancy and bus route network density were used to construct the GWR model, and the variance inflation factor (VIF) of the independent variables test was determined for the two variables (i.e., road area occupancy and bus route network density). VIF = 1.33, i.e., the collinearity between the independent variables was small, but meets the requirements of the regression analysis, and thus, the GWR model-based results are credible.

## Discussion

### Error analysis based on the GWR model

**Figure 5:**
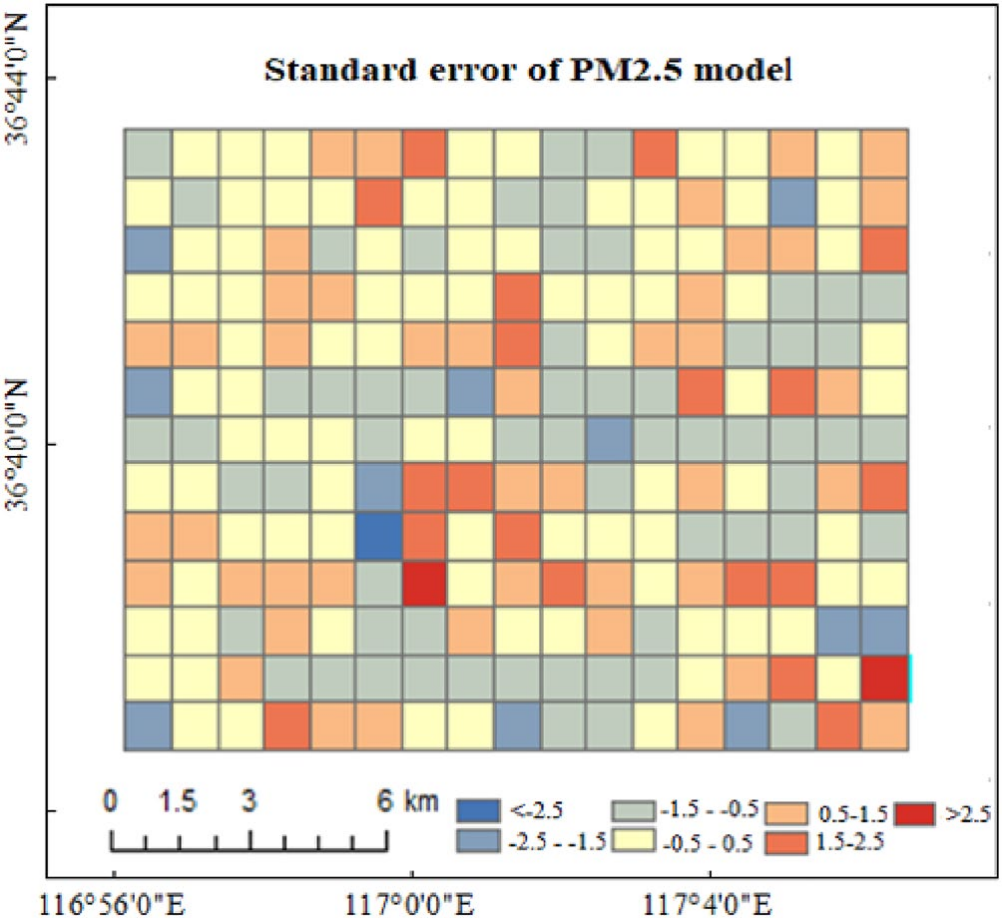
Standard deviation based on the GWR model (The darker the color, the greater the standard deviation). Map created in ArcMap 10.5 of the Environmental System Resource Institute, Inc. (https://www.esri.com/software/arcgis/arcgis-for-desktop).

The standard deviations of the AOD models constructed based on the GWR in the study area is shown in Fig. 5.

**Figure 6:**
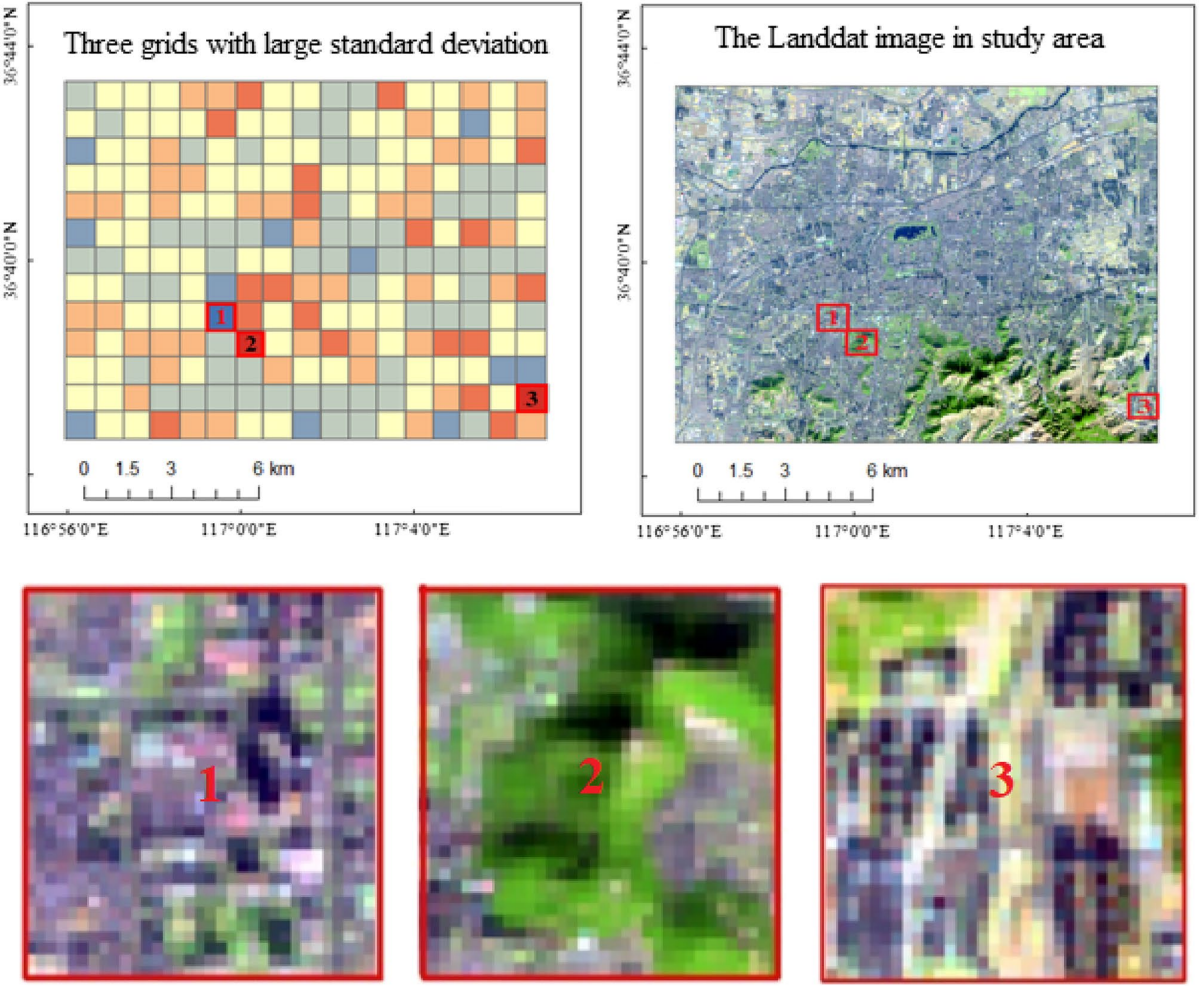
Grids with large standard deviation. There are 3 grids that the standard deviation is greater than 2.5, and the ground cover within the three large standard deviation grids are complex. Map created in ArcMap 10.5 of the Environmental System Resource Institute, Inc. (https://www.esri.com/software/arcgis/arcgis-for-desktop).

As can be seen from Fig. 5^,^ fewer grids had standard deviations of greater than 2.5 or less than -2.5. The AOD model constructed based on GWR has three grids with large standard deviation, Fig. 6.

As can be seen from Fig. 6, the types of ground cover within the three error-prone grids are complex, i.e., a large error exists in the model constructed based on GWR under complex surface conditions. The reasons for this are as follows. The complex surface conditions were prone to lower remote sensing inversion AOD accuracies, leading to large estimation errors. Secondly, for the complex surface conditions, the calculation accuracy of the road network characteristic parameters was lower.

### Spatial–temporal deduction of the remote sensing data

Although remote sensing data can retrieve the spatial distribution characteristics of the AOD (air pollution) well, it is instantaneous data, that is, it can only represent the spatial distribution of the satellite transit time. However, the AOD and traffic exhaust emissions are always changing, so the AOD retrieved by remote sensing has the problem of time scale deduction. To ensure the correspondence of the time scale, the observation data corresponding to the response time of the satellite transit time is generally used for research. In order to reduce the time scale error, in this study, the correlation analysis between the PCDI and the air quality was based on the average value. Therefore, the GWR analysis was conducted using the daily mean value of the AOD and the road network density, road area occupancy rate, intersection number, and bus network density.

The AOD data from MODIS generally contain data for more than three orbits each day (each orbit time is different). The AOD data are commonly missing on days with heavy pollution or very little pollution, e.g., from January 1 to 10, 2017, the air pollution in the study area was serious, and the MCD19A2AOD in Jinan is all null. Thus, the algorithm of the AOD needs to be further improved.

### Scale problem

In addition to spatial heterogeneity, spatial scale is also an important feature of geographical phenomena. Fotheringham et al. developed the multi-scale geographically weighted regression (MGWR) model to study the influence of different scales on air quality. To reduce the impact of the different scales on the analysis, in this study, the gridded study area (i.e., the 1 km grid) was selected to match the 1 km resolution of the AOD product.

**Figure 7:**
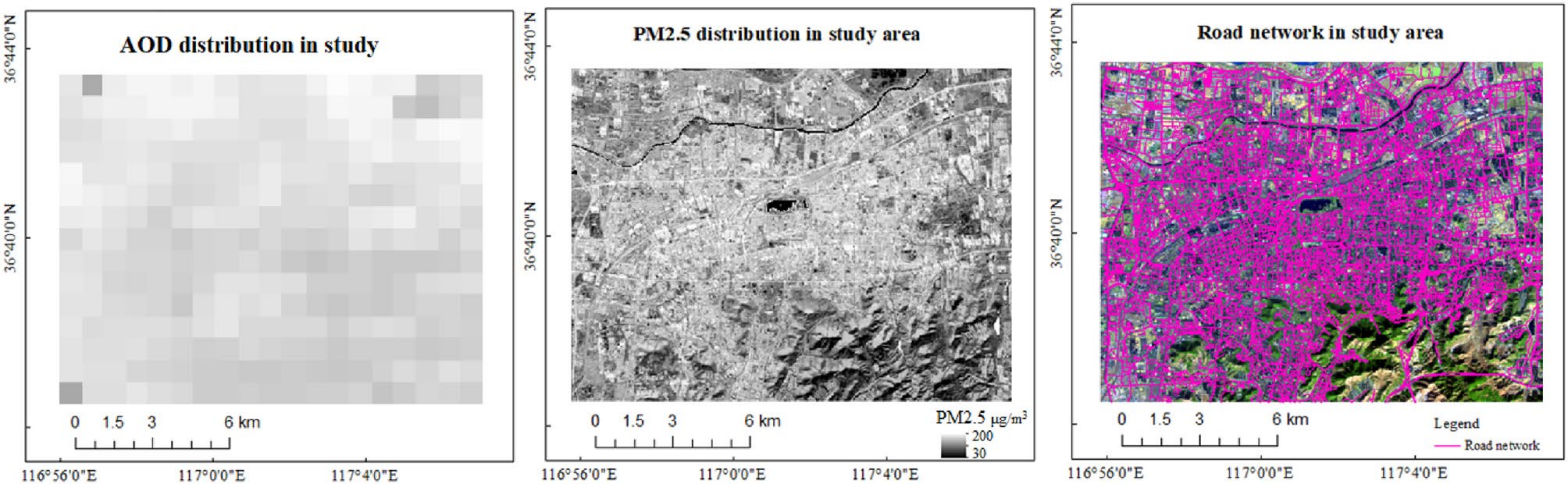
AOD (1000 m), PM2.5(30 m) and road network in the study area. The PM2.5 values along the road network were significantly higher than those in other areas, i.e., the higher resolution can better retrieve the relationships between the roads and air quality. Map created in ArcMap 10.5 of the Environmental System Resource Institute, Inc. (https://www.esri.com/software/arcgis/arcgis-for-desktop).

Haiying et al. constructed the difference index (DI) (red band and near-infrared band based on Landsat8 OLI data to monitor the DI concentration (30 m resolution) (Feng et al. 2018). The AOD (1000 m), DI (30 m) and the road network in the study on December 26, 2017 were shown Fig. 7.

The difference index of the 30 m resolution can better retrieve the relationships between the roads, road network, and air quality, but the difference index of the 30 m resolution lacks a strict theoretical basis, that is, a higher classification of the remote sensing products is a direction for future research.

### Applicability of the model

We used the same method to analyze the data of November 5, 2016, and the result was similar, so this study has better adaptability.

The MCD19A2AOD product was used to analyze the impacts of road traffic characteristic parameters on urban air quality quantitatively, although there is a strong correlation between AOD and air pollutant (PM2.5, PM10, NO_2_, etc.), so the results have some difference with actual air pollutant. In addition, when the weather conditions are bad, remote sensing data cannot be obtained.

The correlations between the PCDI and the AOD were positive, that is, the more congested the traffic, the stronger the relationship between the PCDI and air pollution. When free traffic flow, the contribution rate of vehicle emission to the air pollution is low, and which will cause the accuracy of the model based on GWR reduce. Thus, this study is suitable for traffic congestion environment, and can provide a significant reference for traffic planning and air quality control in congested areas.

## Conclusions

Based on the AOD retrieved from remote sensing data and GWR models, in this study, the impacts of four road network traffic characteristic parameters on air quality were first quantitatively analyzed, including the road network density, road area occupancy, intersection number, and bus network density. The main research conclusions are as follows. There is a strong positive correlation between the PCDI and air quality. Based on the GWR model, AOD has high correlations with the road network density, road area occupancy, intersection number, and bus network density, and these correlations are much higher than ordinary linear regression, that is, GWR refines the local spatial changes in AOD distribution and the road traffic parameters. The correlation between AOD and road area occupancy was the highest. The correlations of AOD with the bus network density and intersections number were both higher than with the road network density, so they have greater impacts on air quality that bus route planning, bus emission reduction, road network planning, and intersection signal timing. The positive correlations of four variables were dominant, and the correlation R^2^ based GWR model all above 0.6. The applicability of the model is limited when the complex surface condition, the bad weather condition, and free traffic flow. The algorithm of the AOD needs to be further improved. This study has certain guiding significance for traffic planning and traffic control, and provides support and basis for traffic planning and control, especially in areas with traffic jam.
